# Supplementary material for: Bimodal fibrosis in a novel mouse model of bleomycin-induced usual interstitial pneumonia
Source: Life Sci Alliance. 2021 Nov 2;5(1):e202101059. doi: 10.26508/lsa.202101059 (PMC8572746; doi:10.26508/lsa.202101059)
Supplement: Supplementary file 1 [file LSA-2021-01059_TableS1.docx]

**Supplemental Materials**

Bimodal fibrosis in a novel mouse model of bleomycin-induced usual interstitial pneumonia

Authors and affiliations

Yoko Miura^1^, Maggie Lam^2^, Jane E. Bourke^2^, and Satoshi Kanazawa^1^

^1^ Department of Neurodevelopmental Disorder Genetics, Nagoya City University Graduate School of Medical Sciences, Nagoya, Aichi, Japan

^2^Department of Pharmacology, Biomedicine Discovery Institute, Monash University, Clayton, Australia

**Table S1 The change of body weight and serum SP-D concentrations with different bleomycin concentrations after BMS administration**

Concentration of Body weight at 1 week Concentration of serum

bleomycin (mg/kg) after BMS (%±S.E.) SP-D (ng/ml ±S.E.)

1.28 93.6 (±1.8) 1425 (±231)

1.6 93.5 (±1.5) 908 (±391)

n=7 for 1.28 mg/kg body weight, n=10 for 1.6 mg/kg body weight. Serum samples were collected at 2 weeks after BMS. There were no significant differences in body weight and serum SP-D.
